# Kras-driven intratumoral heterogeneity triggers infiltration of M2 polarized macrophages via the circHIPK3/PTK2 immunosuppressive circuit

**DOI:** 10.1038/s41598-021-94671-x

**Published:** 2021-07-29

**Authors:** Theodora Katopodi, Savvas Petanidis, Kalliopi Domvri, Paul Zarogoulidis, Doxakis Anestakis, Charalampos Charalampidis, Drosos Tsavlis, Chong Bai, Haidong Huang, Lutz Freitag, Wolfgang Hohenforst-Schmidt, Dimitris Matthaios, Konstantinos Porpodis

**Affiliations:** 1 Department of Medicine, Laboratory of Medical Biology and Genetics, Aristotle University of Thessaloniki, 54124 Thessaloniki, Greece; 2 Department of Pulmonology, I.M. Sechenov First Moscow State Medical University, Moscow, 119992 Russian Federation; 3 Pulmonary Department-Oncology Unit, “G. Papanikolaou” General Hospital, Aristotle University of Thessaloniki, 57010 Thessaloniki, Greece; 4 Third Department of Surgery, “AHEPA” University Hospital, Aristotle University of Thessaloniki, 55236 Thessaloniki, Greece; 5 Department of Anatomy, Medical School, University of Cyprus, 1678 Nicosia, Cyprus; 6 Department of Medicine, Laboratory of Experimental Physiology, Aristotle University of Thessaloniki, 54124 Thessaloniki, Greece; 7 Department of Respiratory and Critical Care Medicine, Changhai Hospital, Second Military Medical University, Shanghai, 200433 China; 8 Department of Pulmonology, University Hospital Zurich, 8091 Zurich, Switzerland; 9 Medical Clinic I, “Fuerth” Hospital, University of Erlangen, 91054 Fuerth, Germany; 10 Oncology Department, General Hospital of Rodos, 85133 Rhodes, Greece

**Keywords:** Cancer, Immunology

## Abstract

Intratumoral heterogeneity in lung cancer is essential for evasion of immune surveillance by tumor cells and establishment of immunosuppression. Gathering data reveal that circular RNAs (circRNAs), play a role in the pathogenesis and progression of lung cancer. Particularly Kras-driven circRNA signaling triggers infiltration of myeloid-associated tumor macrophages in lung tumor microenvironment thus establishing immune deregulation, and immunosuppression but the exact pathogenic mechanism is still unknown. In this study, we investigate the role of oncogenic Kras signaling in circRNA-related immunosuppression and its involvement in tumoral chemoresistance. The expression pattern of circRNAs HIPK3 and PTK2 was determined using quantitative polymerase chain reaction (qPCR) in lung cancer patient samples and cell lines. Apoptosis was analyzed by Annexin V/PI staining and FACS detection. M2 macrophage polarization and MDSC subset analysis (Gr1^−^/CD11b^−^, Gr1^−^/CD11b^+^) were determined by flow cytometry. Tumor growth and metastatic potential were determined in vivo in C57BL/6 mice. Findings reveal intra-epithelial CD163^+^/CD206^+^ M2 macrophages to drive Kras immunosuppressive chemoresistance through myeloid differentiation. In particular, monocytic MDSC subsets Gr1^−^/CD11b^−^, Gr1^−^/CD11b^+^ triggered an M2-dependent immune response, creating an immunosuppressive tumor-promoting network via circHIPK3/PTK2 enrichment. Specifically, upregulation of exosomal cicHIPK3/PTK2 expression prompted Kras-driven intratumoral heterogeneity and guided lymph node metastasis in C57BL/6 mice. Consequent co-inhibition of circPTK2/M2 macrophage signaling suppressed lung tumor growth along with metastatic potential and prolonged survival in vivo*.* Taken together, these results demonstrate the key role of myeloid-associated macrophages in sustaining lung immunosuppressive neoplasia through circRNA regulation and represent a potential therapeutic target for clinical intervention in metastatic lung cancer.

## Introduction

Lung cancer is the primary cause of cancer-related deaths worldwide and is clinically characterized by chemoresistance, poor prognosis and limited therapeutic interventions^1–3^. Recent data indicate that tumor macrophages or tumor-associated macrophages (TAMs), which constitute the majority of immune cells in tumor microenvironment (TME) are linked with poor clinical prognosis and acquired chemoresistance^4,5^. A plethora of studies reveal, TAMs to crosstalk with both stromal and tumor cells. In the TME, supporting the establishment of an immunosuppressive network that favors tumor progression and metastasis^6,7^. However, the exact mechanism of this interaction remains largely unidentified. Growing evidence has indicated that TAMs activity and performance is vastly regulated by myeloid cells, especially myeloid-derived suppressor cells (MDSCs) which are accountable for the intratumoral heterogeneity in lung cancer^8,9^. Furthermore, this intratumoral heterogeneity is also triggered by mutant Kras signaling which too supports tumor-dependent immunosuppression^10,11^.

Circular RNAs (circRNAs) are a class of small noncoding RNAs which have no 5′–3′ polarity and contain no polyA tail^12,13^. CircRNAs are able to regulate macrophage activity and can function as endogenous miRNA sponges or as RNA-binding protein (RBP) agents and control gene expression^14,15^. Many circRNA member have been found to be dysregulated in various types of tumors, including breast lung and gastric cancer^13,16,17^.

In the present study we characterize for the first time the role of circHIPK3/PTK2 in tumor macrophages-related immunosuppression. Our findings show intra-epithelial CD163^+^/CD206^+^ M2 macrophages to trigger Kras-dependent immunosuppressive chemoresistance through MDSCs differentiation. Specifically, monocytic MDSC subsets Gr1^−^/CD11b^−^, Gr1^−^/CD11b^+^ elicited an M2-dependent immune response, creating an immunosuppressive tumor-promoting network via circ HIPK3/PTK2 up regulation. Furthermore, amplification of exosomal circHIPK3/PTK2 expression prompted Kras-driven intratumoral heterogeneity and guided lymph node metastasis in C57BL/6 mice. Overall, these findings reveal a novel crosstalk mechanism between circRNAs and tumor macrophages that promotes lung immunosuppressive metastasis and might be a potential therapeutic target for combating lung cancer metastasis.

## Materials and methods

### Lung cancer tissue samples

Ninety-six NSCLC tissues and paired adjacent non cancerous lung tissues were collected after informed consent from patients in the AHEPA Hospital, Medical School, Aristotle University of Thessaloniki (Thessaloniki, Greece). Tissue samples were collected in the operating room immediately after surgery with non-tumor tissues sent to Pathology for diagnosis by a certified pathologist. Patients received neither chemotherapy nor radiotherapy before tissue collection. The patient study protocol is based on previous studies^18^.

### Cell culture

Human lung adenocarcinoma cells A549 were grown in DMEM, supplemented with 10% fetal bovine serum, 2 mM l-glutamine, 1 mM sodium pyruvate, 100 U/mL penicillin, and 100 μg/mL streptomycin (Biochrom AG, Berlin, Germany). The epithelial lung cancer H1299 and H1975 cells were grown in RPMI-1640 (Invitrogen, Carlsbad, USA) and were supplemented with 10% heat-inactivated fetal calf serum (FCS). Both cell lines were grown to confluence and maintained in a humidified atmosphere at 37 °C and 5% CO_2_.

### Flow cytometry

Macrophages analysis was performed using single cell suspensions, following incubation with antibodies (APC Mouse anti-Human CD206, PE Mouse anti-Human CD163, APC-Cy7 Mouse anti-Human CD80, FITC Mouse anti-Human HLA-DR from BD Biosciences, USA) for 1 h at 4 °C. Cells then were washed three times with flow buffer (5 mL), then centrifuged, and resuspended in 1 mL of flow buffer for analysis. Cell analysis was performed using a FACSCalibur flow cytometer (BD Biosciences, USA). Flow cytometric analysis was performed using the FlowJo software.

### Macrophage polarization

Macrophage polarization was performed according to a previous method^19^. Briefly, THP-1 cells were differentiated into M0 macrophages following incubation with 320 nmol/L PMA for 18 h. In order to produce M1-polarized macrophages, THP-1 cells were treated with 320 nmol/L PMA for 12 h and then cultured with 100 nmol/L PMA plus 100 ng/mL lipopolysaccharide (LPS) and 20 ng/mL IFN-γ for a further 48 h. To generate M2-polarized macrophages, THP-1 cells were treated with 320 nmol/L PMA for 12 h and then cultured with 100 nmol/L PMA plus 20 ng/mL IL-4 and 20 ng/mL IL-13 for a further 48 h.

### Isolation of macrophages from tumor tissues

Freshly isolated patient-derived tumor tissues (biopsies) were cut into pieces and digested in Buffer A containing 0.1 g/L hyaluronidase (Sigma) 1 g/L type 4 collagenase (Worthington), and 0.01 g/L DNase I (Solarbio, China). Cells were then collected and centrifuged at 600 × *g* for 5 min. The pellets were resuspended with ACK Lysing Buffer and washed with Buffer A before filtration with a 100-μm filter. Cells were then collected for further isolation of macrophages and other immune cells. This method was performed according to a previous protocol described in the literature with slight modifications^20^.

### Quantitative real-time PCR analysis

For analysis of mature circRNA expression, 1 μg of total RNA was converted to cDNA using Prime-Script cDNA Synthesis Kit (Takara, Shiga, Japan). Following reverse transcription, qRT-PCR analysis was performed using the SYBR Premix kit (Takara). GAPDH was used as a normalization control for all of the samples. The following primers were used: 5′-AATCGTGGTGAACCCATAGTG-3′ (forward) and 5′-CTGCAAGCATTAGCATCCCT 5′-(reverse) for circPTK2, and 5′-TATGTTGGTGGATCCTGTTCGGCA-3′(forward) and 5′-TGGTGGGTAGACCAAGACTTGTGA-3′ (reverse) for circHIPK3. Complete list of all primers is included in Supplementary Table S3. The qPCR was performed on an ABI Prism 7500 Fast Real-Time PCR system (Applied Biosystems, Foster City, CA, USA) using SYBR Premix Ex Taq II (TaKaRa), and the 2^−∆∆Ct^ method was used to calculate the mRNA expression.

### siRNA targeting circPTK2

The siRNA for circPTK2 knockdown was synthesized by GenePharma (Shanghai, China) to target circPTK2. siRNA was used as a negative control. Briefly, A549 cells were transfected with 80–100 pmol of siRNA using Lipofectamine 2000 (Invitrogen). After 72 h, cells were harvested for qRT-PCR analysis of circPTK2 and linear PTK2 mRNA expression.

### Quantification of cytokines by enzyme-linked immunosorbent assay (ELISA)

Cytokine concentration was measured for each experimental condition by ELISA, using commercial kits purchased from R&D Systems (Minneapolis, MN, USA), according to the manufacturer’s guidelines. The cytokine kits included IFN-γ (DY285), TNF-α (DY210), IL-6 (DY206), IL-10 (DY217B), IL-12 (DY1240) and IL-1β (DY201). Positive controls were included in the ELISA kit.

### Ex vivo tissue culture

Lung tumor samples from consenting Kras (chemoresistant/chemonaive) cancer patients at the AHEPA Hospital Oncology unit were collected, transferred to research lab in cold media, and processed in a sterile tissue culture hood. The culture protocol is modified based on previous studies^21,22^.

### Animals

Six weeks old male C57BL/6 mice were purchased from the Jackson laboratory (Bar Harbor, ME) and were housed under pathogen-free environment with a 12 h light/12 h dark schedule and fed with an autoclaved diet and water ad libitum. The protocol was performed according to a previous study with minor modifications^16^. To establish orthotopic xenografts in mice, A549 (3 × 10^6^) cells transfected with siRNA-NC/si-circPTK2 and directly implanted into the upper region of the lungs. Three days later, mice were randomly divided into four groups. Three separate groups of mice (n = 8) were injected subcutaneously in lung section with PLX-3397 (2 mg/kg body weight in 0.1 mL PBS, once a day and 5 days per week for 8 weeks). Control mice (n = 8) were treated the same with vehicle (0.1 mL PBS). After 4 weeks treatment, mice were sacrificed; lungs and lymph nodes were removed for bioluminescence imaging and quantification of tumor burden. For imaging, mice were given ip injections of 150 mg/kg d-luciferin (Xenogen) 10 min before imaging. To quantitate tumor burden, bioluminescence signals were calculated from the imaging data using the Living Image software 3.2 (Xenogen) according to manufacturer's protocols.

### Statistical analysis

The results are expressed as mean ± SD from at least three separate experiments performed in triplicate. Statistical analysis was performed according to previous methods^23^. The differences between groups were determined with a two-tailed Student’s t-test or ANOVA using Graph Pad software. The results represent the mean ± SD of at least three independent experiments. Differences were considered statistically significant at p < 0.05. Statistically significant data are indicated by asterisks (*p < 0.05, **p < 0.01).

### Ethics approval and consent to participate

The study protocol conformed to the ethical guidelines of the Declaration of Helsinki and was approved by the Institutional Review Board and Ethics Committee of the AHEPA Hospital Medical School, Aristotle University of Thessaloniki.

### Consent for publication

The consent forms were signed by every participant and will be provided upon request.

## Results

### Polarization of CD163^+^ M2 macrophages is associated with mutant Kras status, CD4^+^ T cell ratio, and recurrence free survival

**Figure 1:**
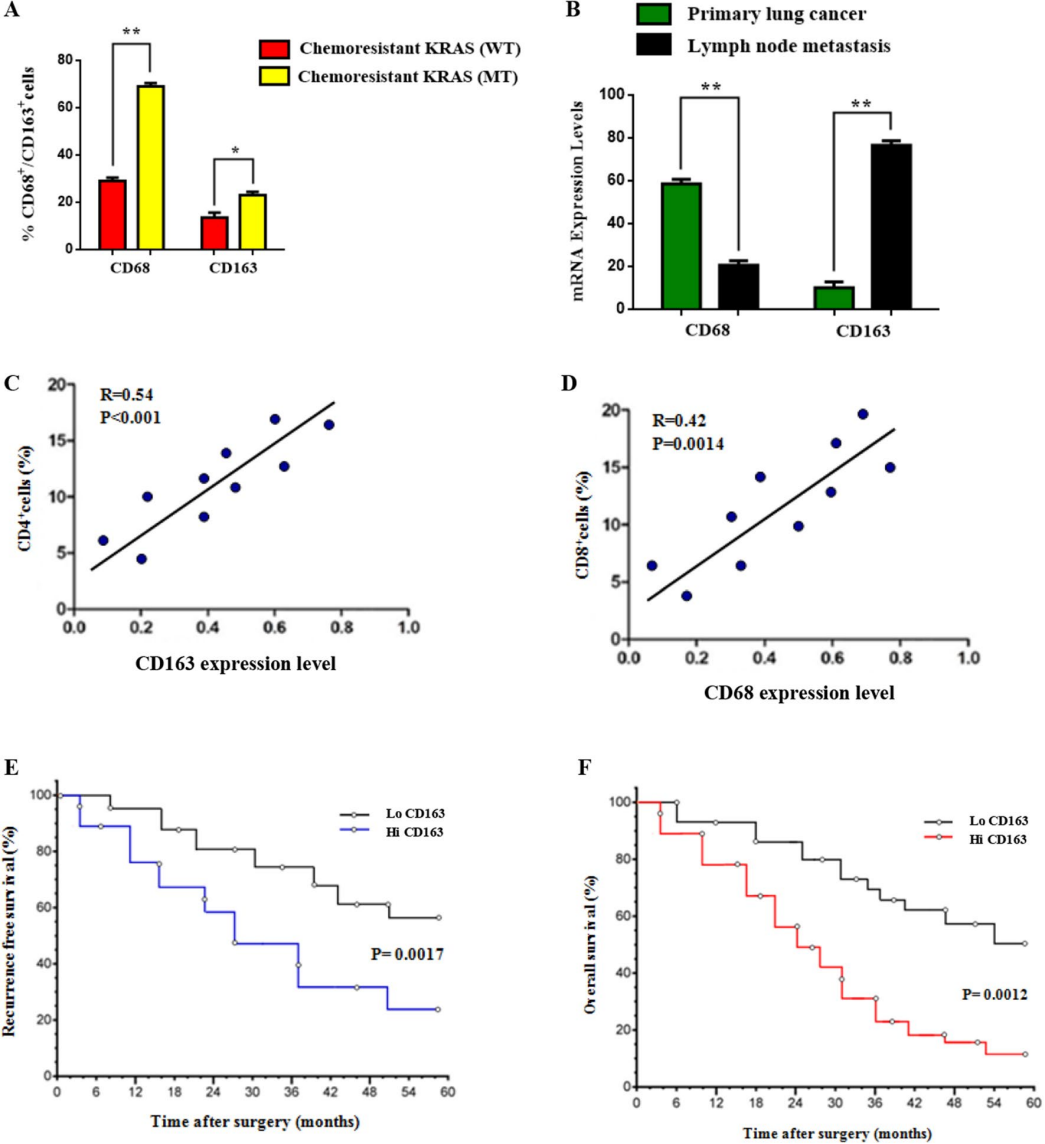
Polarization of CD163^+^ M2 macrophages correlates with mutant Kras status. (**A**) Association of M2 macrophage (CD68^+^/CD163^+^) levels with Kras (WT/MT) chemoresistant patients, using FACS analysis. (**B**) Comparative CD68^+^/CD163^+^ mRNA expression in primary lung cancer or LN metastatic tissues. (**C**,**D**) Association between the CD4/CD8 ratio and M2 macrophage markers CD68^+^/CD163^+^ . The degree of positive correlation was higher in CD4 subset in comparison with CD8 group (R = 0.54 vs. R = 0.42). (**E**,**F**) Percentage of recurrence-free survival (RFS) and overall survival (OS) in LC patients with high or low CD163^+^ expression. The results represent the mean ± SD of three independent experiments. Statistically significant data are indicated by asterisks (*p < 0.05, **p < 0.01).

To evaluate the clinical significance of M2 macrophages in lung tumor development and effector phenotype acquisition, the expression of TAMs markers (CD68, CD163) in tissue sections from chemoresistant Kras patients was assessed. Statistical analysis revealed an increased percentage of CD68^+^/CD163^+^ TAMs to be correlated with depleted T cell levels and immune impediment in chemoresistant Kras (MT) patients (Fig. 1A, Supplementary Table S4). In addition, near the tumor invasive front, relative high levels of CD163^+^ were detected in LN metastatic Kras patients in advanced stage (Stage III–IV) compared to chemonaive-responding to chemotherapy regimen-early stage individuals, indicating a CD163^+^/M2 macrophage involvement in tumor propagation (Fig. 1B). Next, we investigated the association between CD163 and CD68 expression with T cell ratio from the peripheral blood of included patients. Findings reveal, a positive correlation between the CD4^+^/CD163^+^ and CD8^+^/CD68^+^ T cell ratio (Fig. 1C,D) which can be attributed to cross talking between macrophage surveillance and T cell signaling in tumor microenvironment. These T cells/M2 associations in lung epithelium play a key role in clinical prognosis and response to immunotherapeutic approaches. To better understand this mechanism, the clinical significance of CD163 expression in patient overall survival (OS) and recurrence free survival (RFS) analysis was investigated. Prognostic analysis showed that, at the invasive border, upregulated levels of CD163 expression were significantly associated with worse RFS (P = 0.0017). Kaplan–Meier surviving curves were employed for the analysis of the prognostic potential of CD163 in LC patients. Data show that low CD163 intensity was closely associated with better patient prognosis (Fig. 1E,F). Overall, these results indicate the crucial role of CD163 + TAMs at invasive front which promote tumor expansion and affect lung cancer patients survival.

### Intra-epithelial CD163^+^/CD206^+^ M2 macrophages drive Kras immunosuppressive chemoresistance

**Figure 2:**
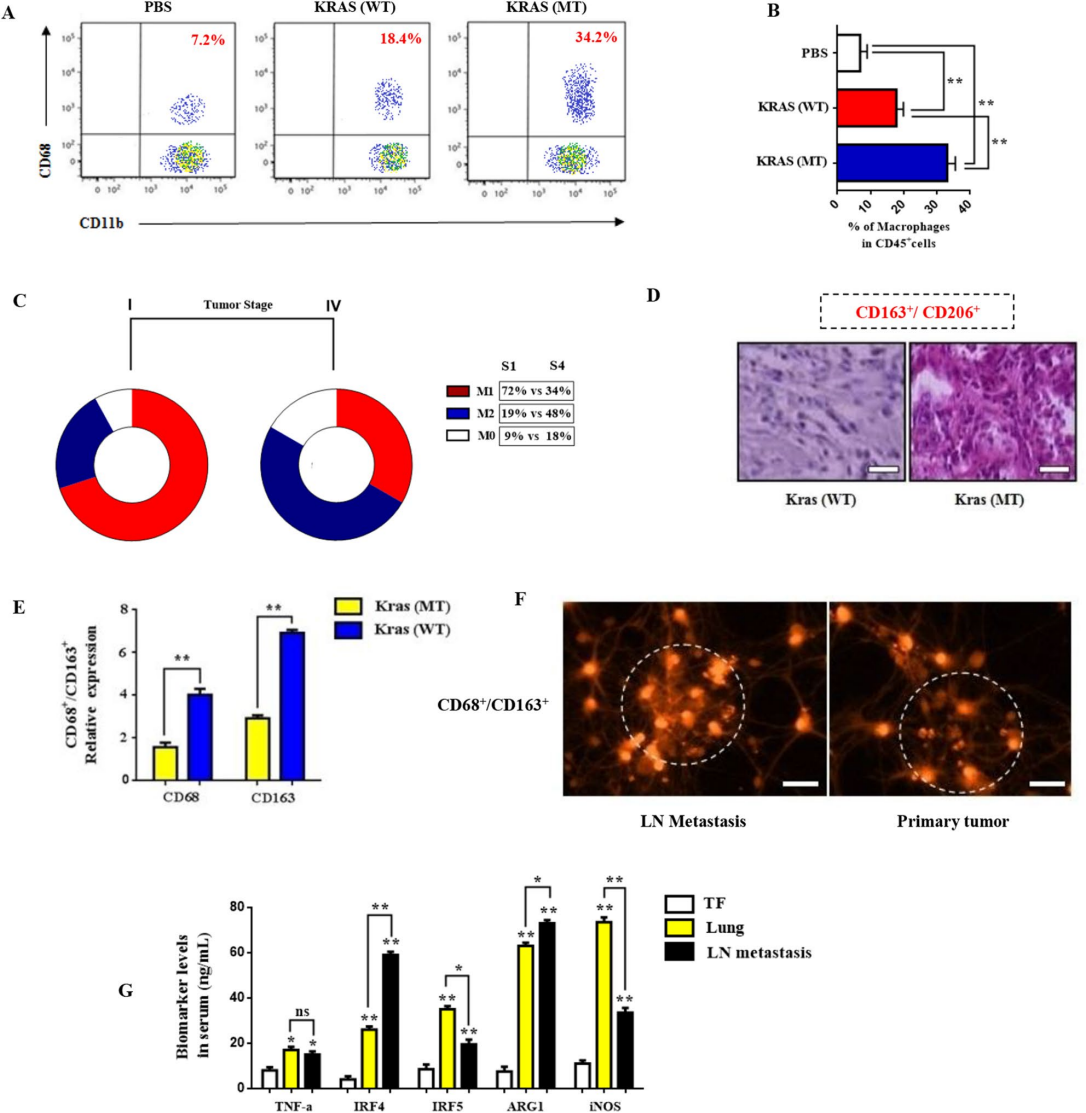
Intra-epithelial CD163^+^/CD206^+^ M2 macrophages prompt Kras immunosuppressive chemoresistance. (**A**,**B**) Quantification of CD11b^+^CD68^+^ macrophages in Kras patient tissue samples using FACS analysis. The results represent the mean ± SD of three independent experiments. (**C**) Distribution percentage of macrophage subsets (M1/M2/M0) in lung tissues from primary Stage I and metastatic Stage IV patient groups. (**D**,**E**) Immunohistochemical analysis of CD68^+^/CD163^+^ expression in Kras (WT) and Kras (MT) groups. The results represent the mean ± SD of three independent experiments. (**F**) Immunofluorescene analysis of CD68^+^/CD163^+^ positive cells among intratumoral macrophages (magnification × 200). Images were captured using Carl Zeiss fluorescence confocal microscope. (**G**) Relative expression of M1/M2 biomarkers TNF-a, IRF4, IRF5, ARG1 and iNOS, in intratumoral and peripheral macrophage subsets isolated from PBMCS and tissue biopsies from tumor free (TF), LC and LN metastasis groups respectively. Data represent the mean ± SD of three independent experiments (*p < 0.05; **p < 0.01). Differences were considered statistically significant at p < 0.05. Statistically significant data are indicated by asterisks (*p < 0.05, **p < 0.01).

To better comprehend the Kras-induced M2 macrophage response, the infiltration of macrophage cellular subsets in lung TME was evaluated. Recent reports provide evidence that certain macrophage subsets can regulate fate of myeloid differentiation, whereas others contribute to immunosuppression maintenance in tumor microenvironment^24,25^. To validate this distribution of macrophages in tumor stroma, we analyzed the expression of macrophage marker CD68 in patient derived tissues. We found higher density of stromal macrophages in Kras (MT) tissues than in para-cancer or wild type Kras samples (Fig. 2A,B). To further characterize the immunological profiles of these distinct patient groups we analyzed the dispersal of macrophage subsets in lung tissues. Importantly, the percentage of M1 macrophages (CD68^+^/CD86^+^) was upregulated in patients from the Kras (WT) group compared to low CD163^+^/CD206^+^ M2 ratio in Kras (MT) group (Fig. 2C). Furthermore, the levels of M0 (F4/80^+^) macrophages where increased during late metastatic stages. Next, in order to further characterize the M2 cell subsets present in lung tumors we compared the profile of CD163^+^/CD206^+^ cells isolated from chemoresistant patients with lung or lymph node metastasis with wild type (WT) or mutant (MT) Kras status (Fig. 2C,D). Interestingly, increased CD68^+^/CD163^+^ expression was closely associated with low T to MDSC immune infiltration in Kras (MT) patients (Fig. 2D,E). Additionally, CD68^+^/CD163^+^ expression was significantly higher in intratumoral LN metastatic regions of Kras patients (MT) indicating a M2-related immunosuppressive phenotype (Fig. 2F). Furthermore, the mRNA expression of distinctive M2 markers (Arg1, IRF4) was increased, in metastatic lymph node tissues, whereas the expression of M1 markers (IRF5, TNF-a, iNOS) was restricted in early stage lung cancer samples (Fig. 2G), indicating T cell immunity suppression at a more advanced disease stage.

### Monocytic MDSC subsets Gr1^−^/CD11b^−^, Gr1^−^/CD11b^+^ trigger M2 macrophage enrichment

**Figure 3:**
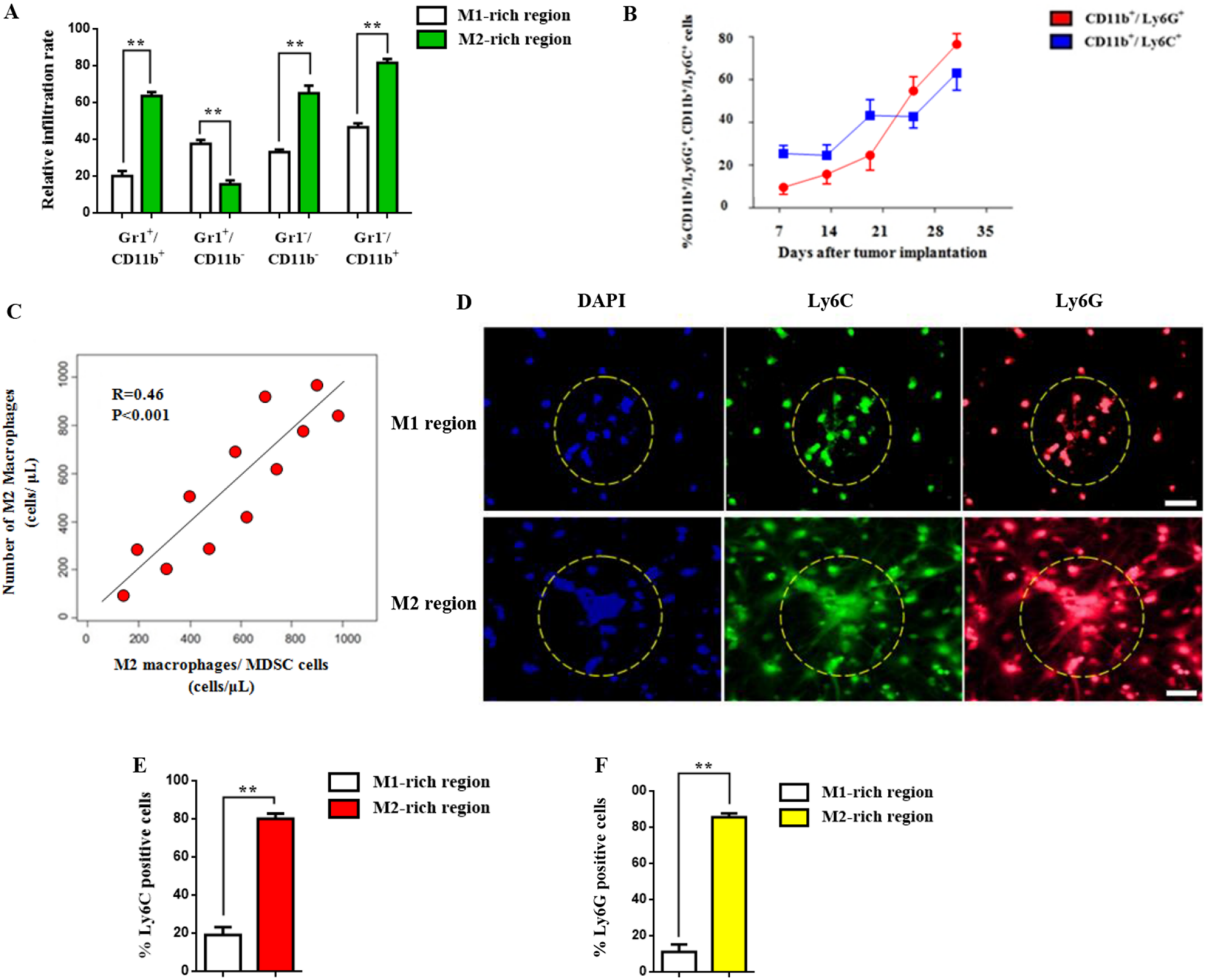
Monocytic MDSC subsets Gr1^−^/CD11b^−^, Gr1^−^/CD11b^+^ elicit M2 macrophage enrichment. (**A**) Relative infiltration rate of MDSC subsets in M1/M2 macrophage regions. MDSC infiltration was evaluated by the presence of gMDSC (Gr^−^/CD11b^−^) and mMDSC (Gr^−^/CD11b^+^) cells in primary and metastatic tissues. (**B**) Induction and infiltration of mMDSCs (CD11bLy6C+) and gMDSCs (CD11b + Ly6G+) in, lung and lymph node tumor. A549 cells (2 × 106) were orthotopically implanted in C57BL/6 mice. Monitoring of MDSCs infiltration started three days post impantation. (**C**) Graph showing positive correlation between the M2/MDSC ratio and M2 macrophage cell numbers. (**D**) Immunofluorescence analysis of Ly6C/Ly6G positive cells in mice bearing A549 tumors (magnification × 200). Confocal microscopy images of Ly6C (green), Ly6G labeled cells (red) and DAPI (blue). Images were captured using Carl Zeiss fluorescence confocal microscope. (**E**,**F**) Data represent the mean ± SD of three independent experiments (*p < 0.05; **p < 0.01). Differences were considered statistically significant at p < 0.05. Statistically significant data are indicated by asterisks (*p < 0.05, **p < 0.01).

To further investigate the mechanism by which M2 macrophages establish lung tumoral immunosuppression we examined the granulocytic/monocytic MDSC expression in lung biopsies. Induction and infiltration of mMDSCs and gMDSCs in M1/M2 regions was measured using FACS analysis. Specifically, MDSC infiltration in M2-rich regions was characterized by the presence of Gr^**−**^/CD11^**−**^ and Gr^**−**^/CD11b^**+**^ monocytic MDSC cells whereas Gr^**+**^/CD11b^**−**^ gMDSC levels were significantly upregulated in M1 tumoral sections indicating a potential link (Fig. 3A). To better understand this myeloid differentiation and the role of immunosuppression-related expansion of heterogeneous MDSC subsets we investigated mMDSCs and gMDSCs infiltration up to five weeks post A549 tumor implantation. Pulmonary mMDSC infiltration was reduced compared to gMDSC infiltration in primary tumor. In addition, lymphatic gMDSC infiltration was increased 34% by week 3 preceding the metastatic lung lesions detection (Fig. 3B). Furthermore, a positive correlation (R = 0.46, p < 0.001) between the M2/MDSC ratio and M2 cell levels was observed which can be attributed to MDSC differentiation towards a M2 phenotype in lung tumor microenvironment (Fig. 3C). Likewise, M2 macrophages enrichment was characterized by the presence of Ly6G/Ly6C positive cells and their expression was restricted to the invasive edge of M2 rich tumor regions (Fig. 3D,E). However, in M1 rich lesions Ly6G/Ly6C expression was decreased significantly (Fig. 3E,F) followed by co-localization of Ki67 positive tumor cells with MDSCs.

### Enhanced circHIPK3/PTK2 expression prompts Kras-driven intratumoral heterogeneity

**Figure 4:**
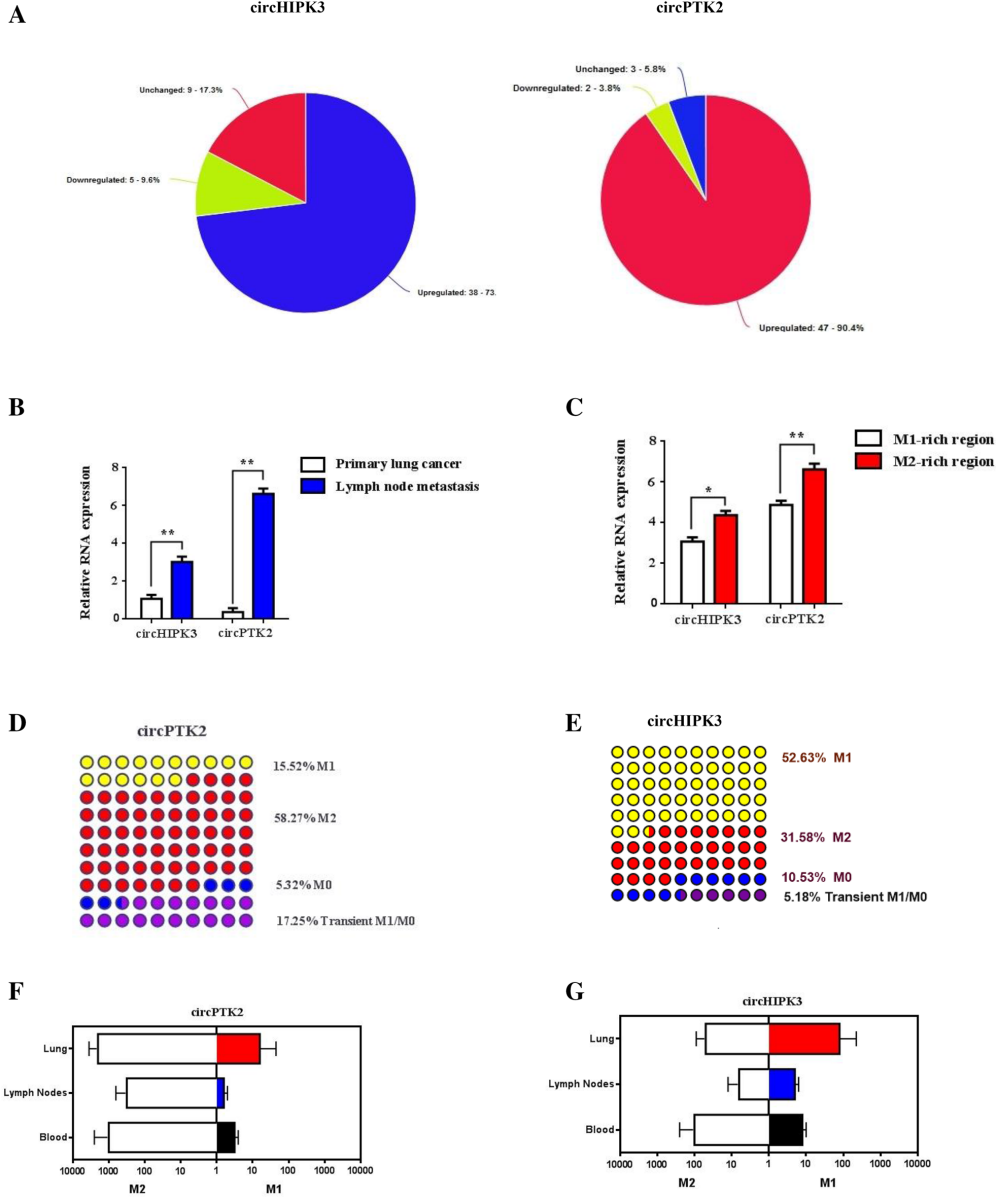
Augmented circHIPK3/PTK2 expression sustains Kras-driven intratumoral heterogeneity. (**A**) Expression profiles analysis of circHIPK3, circPTK2 in paired samples of NSCLC/Kras and adjacent non tumoral tissues by qRT-PCR. (**B**) qRT-PCR analysis of circHIPK3, circPTK2 expression in primary lung cancer and metastatic LN tissues. (**C**) Relative RNA expression of circHIPK3, circPTK2 isolated from tumor invasive front (M1-rich region) and stroma (M2-rich region) tissues. (**D**,**E**) Comparison percentage analysis of circHIPK3, circPTK2 levels in macrophage subsets (M0/M1/M2) from NSCLC patients, using qRT-PCR. (**F**,**G**) Relative degree of circHIPK3/circPTK2 enrichment in macrophage subpopulations isolated from lung, lymph nodes and blood respectively. Differences were considered statistically significant at p < 0.05. Statistically significant data are indicated by asterisks (*p < 0.05, **p < 0.01).

Recent studies report the dysregulation of circular RNAs (circRNAs) to trigger M2 polarization and prompt macrophage-related chemoresistance^26,27^. To access this, we investigated the levels of two distinctive circRNAs, circHIPK3 and circPTK2, which play an essential role in lung cancer progression and development^28,29^. To do so, we performed expression profiles analysis of circHIPK3, circPTK2 in 52 paired samples of NSCLC/Kras and adjacent non tumorous tissues by qRT-PCR. Results reveal that circPTK2 was significantly upregulated in 90.4% (47/52) of the NSCLC tissues examined compared to the levels of circHIPK3 (73.1%, 37/52) from matched adjacent non tumor tissues (Fig. 4A,B). To evaluate the clinical significance of these dysregulated circRNAs in Kras oncogenic signaling, we compared their expression in primary and metastatic Kras (MT) tumor samples. Interestingly, circHIPK3 is expressed in both Kras groups, whereas circPTK2 is highly present in metastatic LN tissues (Fig. 4C). Since circRNAs affect M2 polarization we investigated their role in macrophage differentiation. Intriguingly, we found that circPTK2 is mainly expressed at the tumor invasive front (M1-rich region) and stroma (M2-rich region), while circHIPK3 expression is mainly M2-related and is located into the tumor nest and the invasive periphery of tumor (Fig. 4D). To better estimate the macrophage-dependant circRNA crosstalk, we estimated the three subpopulations of macrophages M0, M1, and M2 using FACS analysis in NSCLC patients. Result analysis of the macrophage characterization showed that pro-tumoral M2 macrophages were more enriched in circPTK2 rather than those with circHIPK3 (58% vs. 32%) (Fig. 4D,E). On the contrary M1 and M0 macrophages co-expressed augmented levels of circHIPK3 (M1: 53% vs. 16%, M0: 11% vs. 5%) whereas, circPTK2 was traced on transient M0/M1 phenotype macrophages (17% vs. 6%) (Fig. 4D,E). Given that infiltrating tumor macrophages (mainly M2 subtype) are largely responsible for tumor progression and metastasis, we assessed circHIPK3/circPTK2 enrichment in macrophage subpopulations from lung, lymph nodes and blood respectively. To our surprise, circHIPK3 was co-expressed mutually in M1/M2 macrophages mainly in lungs and blood indicating its involvement in macrophage-related tumorigenesis (Fig. 4F,G). On the contrary, circPTK2 enrichment in mainly M2-dependent and is located both in primary (lungs) and metastatic (lymph nodes, blood) regions.

### Co-inhibition of circPTK2 and M2 macrophages reduces tumor volume and metastatic foci development

**Figure 5:**
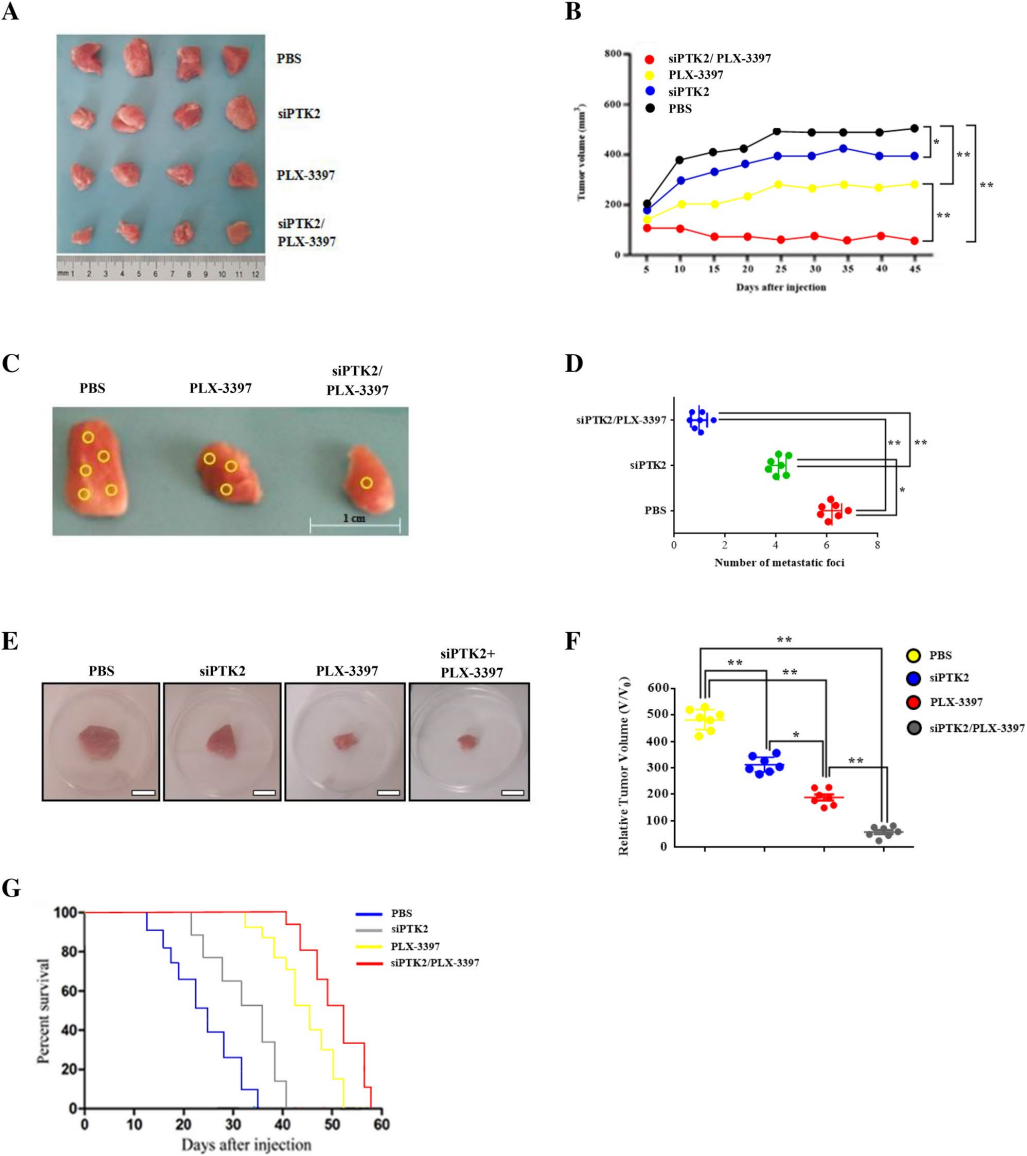
Co-inhibition of circPTK2 and M2 polarization reduces tumor volume and metastatic foci development. (**A**) Characteristic images of excised tumors from mice following si-circPTK2/PLX-3397 treatment. (**B**) Tumor volume analysis of mice treated at various groups. (**C**) In vivo metastatic analysis of lymph node metastasis from si-circPTK2/PLX-3397-treated groups. Images show representative lymph node metastatic foci highlighted in yellow from different groups. (**D**) Statistical analysis of the number of metastatic foci of each group. (**E**) Representative photographs of excised tumors from C57BL/6 mice after intravenous treatment with si-circPTK2/PLX-3397. (**F**) Relative tumor volumes (V/V0) of A549 tumor bearing mice following intravenous administration. (**G**) Survival rates of tumor-bearing mice after a 60-day tumor challenge in each group. Data were given as the mean ± SD (n = 6) (*p < 0.05, **p < 0.01). Data represent the mean ± SD of three independent experiments.

To comprehend the tumorigenic role of circPTK2-dependent M2 polarization in lung cancer, we used a tumor mouse model. To do so, we employed specifically designed siRNA targeting circPTK2 along with the M2 macrophage inhibitor PLX-3397. Initially, A549 cells were stably infected with si-circPTK2 or si-NC, which were then injected subcutaneously into C57BL/6 mice. Three weeks post injection, the tumors were removed, weighted and photographed. Analysis of tumor formation revealed a substantial reduction in lung tumor volume of si-circPTK2 -treated mice compared to PBS-treated mice (Fig. 5A). In addition, co-treatment with si-circPTK2/PLX-3397, considerable inhibited tumor growth in comparison with single group treatments (siPTK2 vs. PLX-3397) or control group (PBS) (Fig. 5B). Furthermore, tumors derived from circPTK2/PLX-3397 knockdown clones developed at significantly slower rates than control tumors. Next, we investigated the effect of circPTK2 inhibition on secondary metastasis by using an vivo metastasis assay. Interestingly, we observed a decreased number of lymph node metastatic foci in circPTK2/PLX-3397 group compared with PLX-3397-treated mice (Fig. 5C,D). In order to understand this observed tumor suppression efficacy, intravenous administration of circPTK2 or PLX-3397 was performed in A549-tumor bearing mice. Relative tumor volume assessment revealed significantly higher rates of tumor reduction for circPTK2/PLX-3397 treatment compared to controls (Fig. 5E,F). This was followed by, tumor volume shrinkage and depleted Ki-67 antigen expression in NCs-treated groups. Mice injected with siPTK2 alone had a significantly shorter median overall survival (OS) after tumor implantation (35 days) than mice injected with the siPTK2/PLX-3397 (median OS, 56 days) (Fig. 5G). Thus, these results confirm that PTK2/M2 signaling pathway inhibition restrains lung tumor growth followed by a decrease in metastatic potential which extends survival in vivo.

### Exosomal expression of circPTK2 triggers an immunosuppressive metastatic circuit

**Figure 6:**
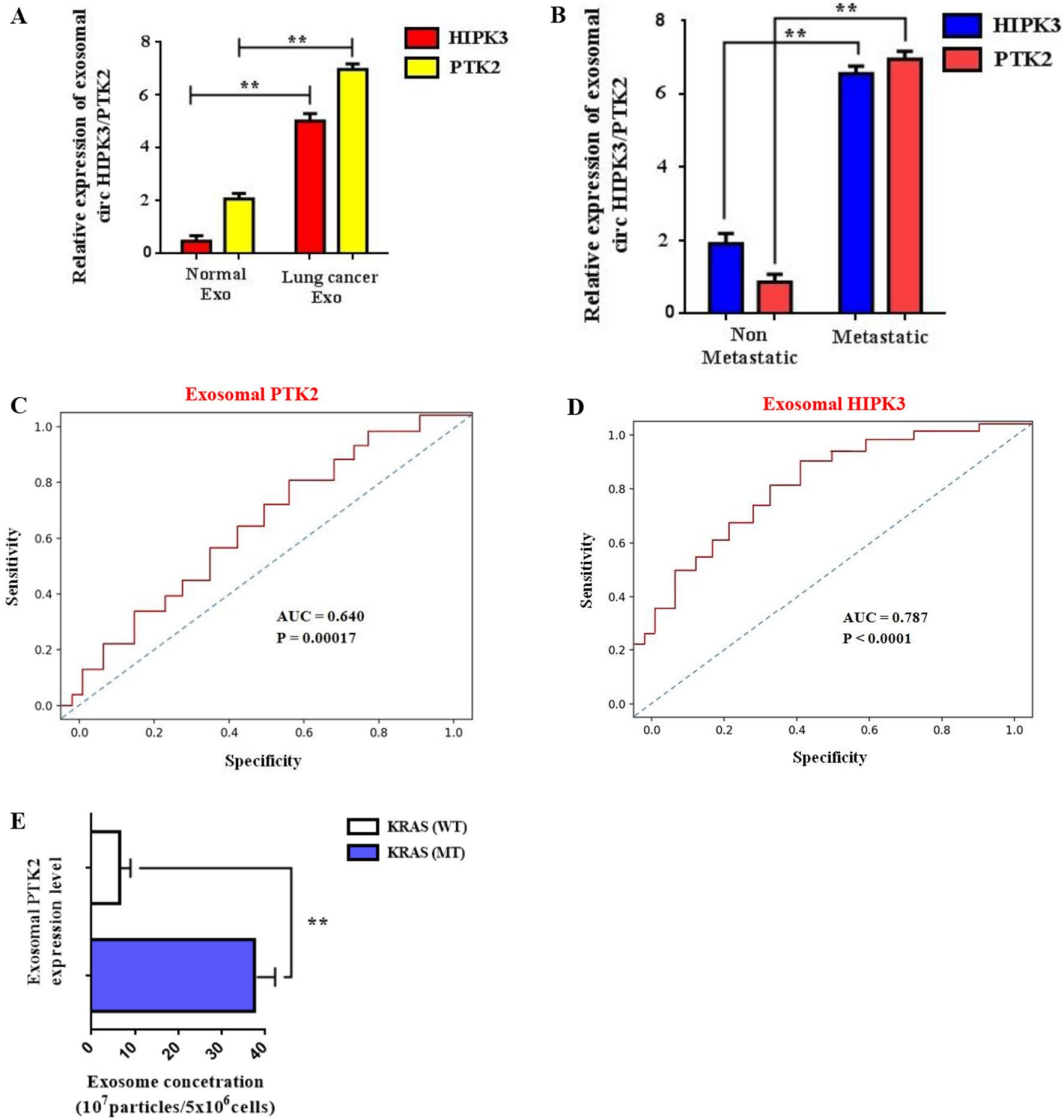

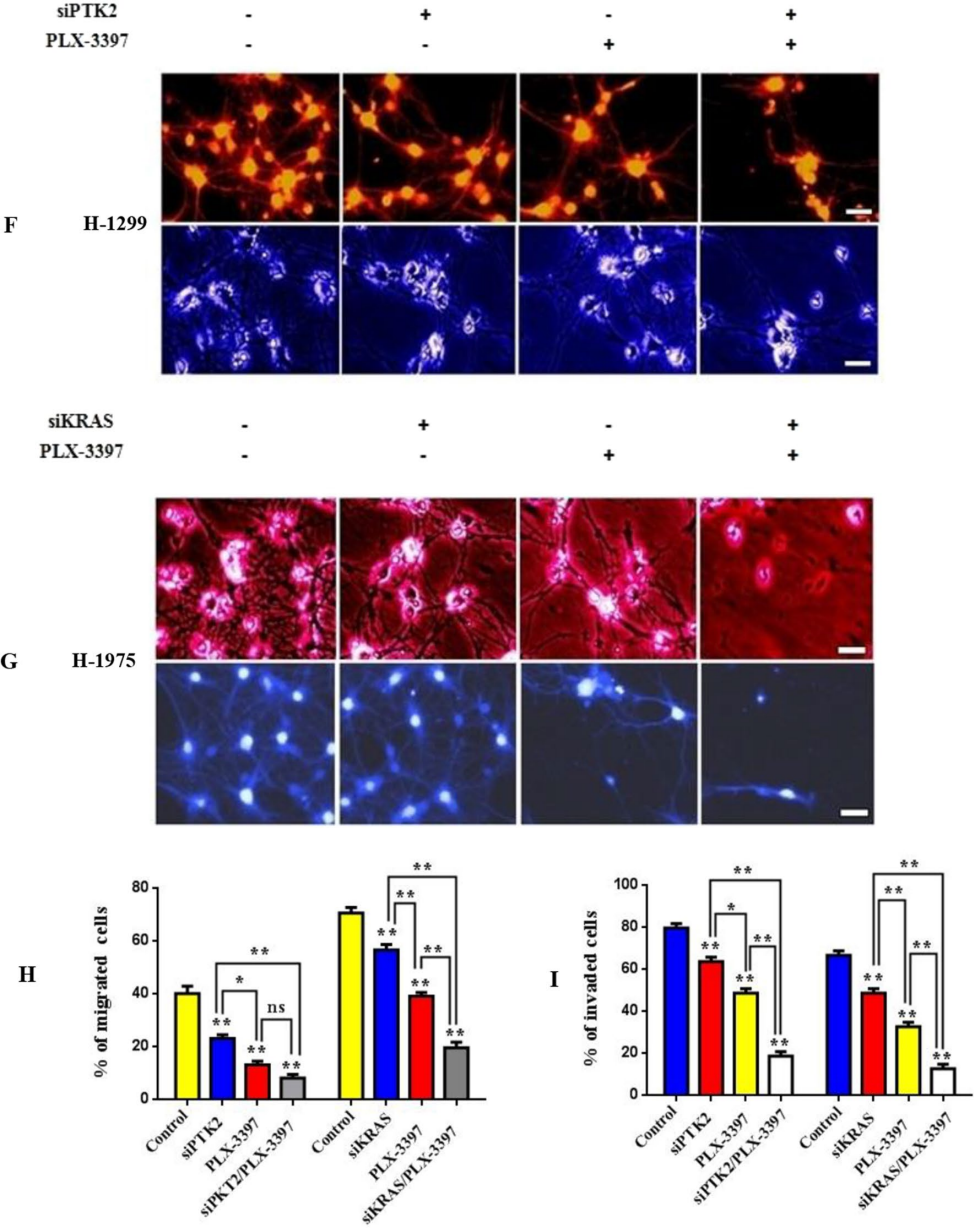
Exosomal expression of circPTK2 triggers an immunosuppressive metastatic circuit. (**A**) Relative exosomal levels of circHIPK3, circPTK2 isolated from patient-derived serum, were measured using RT-PCR. (**B**) Exosomal serum circPTK2/circHIPK3 expression from metastatic (Stage III–IV) and non metastatic (Stage I–II) NSCLC patient groups. (**C**,**D**) ROC curve analysis and AUC values for circPTK2 and circHIPK3 respectively. Data represent the mean ± SD of three independent experiments (*p < 0.05; **p < 0.01). (**E**) Quantified exosomal circPTK2 expression from Kras (WT) and Kras (MT) patient groups. (**F**) Representative images from migration and invasion assays of H1299 cells after transfection with siRNA against circPTK2 (4 μg) and PLX-3397 M2 inhibitor (25 μΜ) for 24 h. (**G**) Representative images from migration and invasion assays of H1975 cells after transfection with siRNA against Kras (4 μg) and PLX-3397 M2 inhibitor (25 μΜ) for 24 h. (**H**,**I**) Quantitative analysis of migration and invasion assays following cell transfection. The results represent the mean ± SD of three independent experiments. Differences were considered statistically significant at p < 0.05. Statistically significant data are indicated by asterisks (*p < 0.05, **p < 0.01).

Latest studies portray circRNA expression to be vastly regulated by exosomes present in lung tumor microenvironment that establish an immunosuppressive niche^30–32^. To verify this, we investigated the differential expression of circHIPK3, circPTK2 in patient-derived serum exosomes. Exosomes were collected from blood of 72 NSCLC patients and 76 healthy donors. Findings show exosomal circPTK2 to be highly expressed in lung cancer patients in comparison with circHIPK3 levels and healthy individuals (Fig. 6A). Furthermore, increased exosomal serum circPTK2/circHIPK3 expression was found in metastatic (Stage III–IV) NSCLC patients compared with the non metastatic (Stage I–II) group indicating an association of exosomal circPTK2/circHIPK3 with lymph node metastasis (Fig. 6B). To better comprehend the circPTK2/circHIPK3 metastatic potential we employed AUC and ROC curves to evaluate the specificity and sensitivity of these circRNAs. Data analysis reveal that in metastatic tissues, the AUC values were 0.640 and 0.787 for circPTK2 and circHIPK3 respectively (Fig. 6C,D). Furthermore, increased exosomal circPTK2 expression was observed in Kras (MT) patients rather than the Kras (MT) group (Fig. 6E). These findings indicate the high clinical sensitivity and specificity of exosomal circPTK2/circHIPK3 which can be used as blood detection index for diagnosis of advanced metastatic cancer in NSCLC patients. To further validate the role of circPTK2 signaling cascade in metastatic potential in tumor stroma were treated metastatic H1299 and H1975 lung cells with si-circPTK2 and Kras/M2 inhibitors and subjected them to invasion or migration analysis respectively (Fig. 6F,G). Co-treatment with siPTK2/PLX-3397 inhibitor significantly reduced the percentage of migrated cells compared with single siPTK2 or PLX-3397 treatments (Fig. 6H). Conversely, cells transfected with siKras/PLX-3397 inhibitors displayed an inferior reduction in migration rates in comparison with siKras inhibitor group or single treatments. Furthermore, cell invasiveness was drastically confined in the siKras/PLX-3397 group (Fig. 6I), whereas co-treatment with siPTK2/PLX-3397 inhibitor reduced the percentage of invaded cells in a lower rate.

## Discussion

**Figure 7:**
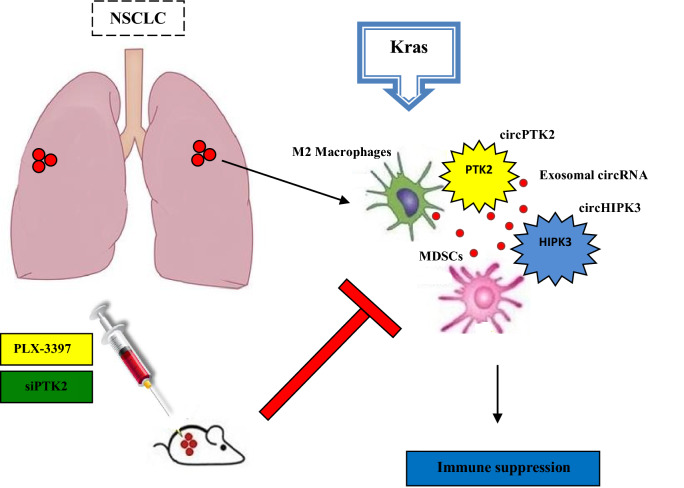
The molecular mechanism of Kras-induced immunosuppression via infiltration of intra-epithelial CD163^+^/CD206^+^ M2 macrophages and circHIPK3/PTK2 enrichment.

Gathering data reveal the crucial role of circRNA signaling in tumor progression and development^33,34^. In our study we describe for the first time the molecular mechanism of interaction between CD163^+^ M2 macrophages and circHIPK3/PTK2 axis which promotes lung intratumoral immunosuppression (Fig. 7). Our findings provide a biochemical explanation for the clinical association between increased circHIPK3/PTK2 expression and metastatic progression in NSCLC. Recently, circPTK2 was also associated with tumor growth and metastasis in colorectal cancer (CRC) and promoted EMT of CRC cells in vitro and in vivo by binding to vimentin protein on sites Ser38, Ser55 and Ser82^35^. Furthermore, circPTK2 is also differentially expressed in bladder carcinoma and promotes the proliferation and migration of bladder tumor cells^29^. In a similar manner, circHIPK3 modulates autophagy via MIR124-3p-STAT3-PRKAA/AMPKα signaling in STK11 mutant lung cancer cells^36^. CircHIPK3 was also found to promote oxaliplatin-resistance in colorectal cancer through autophagy by activating the downstream Bcl-2/beclin1 signaling pathway^37^. Immature myeloid cells (IMC) in tumor microenvironment can also establish chemoresistance via inflammatory cytokine production and inhibition of lymphocyte homing^8,9^. In our study, we found monocytic MDSC subsets Gr1^−^/CD11b^−^, Gr1^−^/CD11b^+^ to be associated with Kras-related chemoresistance via M2 macrophage enrichment. Likewise, exosomes produced by mesenchymal stem cells, drive differentiation of myeloid cells into immunosuppressive M2-polarized macrophages with enhanced l-Arginase activity and IL-10 secretion in breast cancer^38,39^. In addition, AMPKα1 regulates the immunosuppressive activity and differentiation of tumor-MDSC by regulating the differentiation of monocytic-MDSC (mMDSC) to macrophages, suggesting AMPK inhibition as a potential therapeutic strategy to restore protective myelopoiesis in cancer^40^. Immunosuppression can also be established through exosomes secreted by immune or tumor cells inside the tumor stroma^41,42^. Our findings reveal an association of exosomal circPTK2/circHIPK3 expression with lymph node metastatic immunosuppression. Equally, circRNA-002178 can act as a ceRNA to promote PDL1/PD1 expression via sponging miR-34 in cancer cells to induce T-cell exhaustion in lung adenocarcinoma^43^. Human mutant p53 cancer cells can also reprogram macrophages to TAMs via miR-1246-enriched exosomes. These p53-reprogrammed TAMs favor anti-inflammatory immunosuppression with increased activity of TGF-β^44^. In summary, we identify a critical role for circPTK2/circHIPK3 in monocytic MDSC differentiation into CD163^+^ M2 macrophages.

## Conclusions

Taken together, this study demonstrates the key role of circRNAs circPTK2/circHIPK3 in Kras-related lung cancer progression through infiltration of CD163^+^/CD206^+^ M2 macrophages and monocytic MDSC subset (Gr1^−^/CD11b^−^, Gr1^−^/CD11b^+^) recruitment. These findings reveal a novel crosstalk mechanism between circRNAs and tumor macrophages that promote Kras-driven lung immunosuppressive metastasis and can serve as a potential therapeutic target for NSCLC.

## Supplementary Information

Supplementary Information.

Supplementary Table S1.

Supplementary Table S2.

Supplementary Table S3.

Supplementary Table S4.

## Data Availability

All data generated or analyzed during this study are available from the corresponding author upon reasonable request.

## Abbreviations

TAMs: Tumor-associated macrophages
TME: Tumor microenvironment
MDSCs: Myeloid derived suppressor cells
circRNA: Circular RNA
RFS: Recurrence free survival
FOXP3: Forkhead box P3
IMC: Immature myeloid cells
IRF4: Interferon regulatory factor 4
LAG-3: Lymphocyte-activation gene 3
AMPK: 5′ Adenosine monophosphate-activated protein kinase
GATA-3: GATA binding protein 3
