# Supplementary material for: Kras-driven intratumoral heterogeneity triggers infiltration of M2 polarized macrophages via the circHIPK3/PTK2 immunosuppressive circuit
Source: Sci Rep. 2021 Jul 29;11:15455. doi: 10.1038/s41598-021-94671-x (PMC8322174; doi:10.1038/s41598-021-94671-x)
Supplement: Supplementary file 1 — Supplementary Information. [file 41598_2021_94671_MOESM1_ESM.pdf]

Supplementary Methods

Reagents

K-Ras (G12C) inhibitor was purchased from Sigma (Sigma, Germany, Europe). Polyclonal antibodies to phospho-AKT (Ser-473), phospho-AKT (Thr-308), phospho-ERK1/2 (Thr-202/Tyr-204), phospho-p38 (Thr-180/Tyr-182), ERK1, p38, phospho-JNK1/2 (Thr-183/Tyr-185), and N-cadherin were obtained from Cell Signaling Technology (Beverly, MA). Monoclonal antibodies for CD68, CD163, SMARCE1, TNF-a, IRF4, IRF5, ARG1 and iNOS, were purchased from Santa Cruz Biotechnology (Santa Cruz, CA). Rabbit polyclonal antibodies for BACH2, GATA3, BAX and PUMA were purchased from Abcam (Cambridge, UK). The macrophage M2 inhibitor PLX-3397(Pexidartinib) was purchased from SelleckChem (Cambridge, UK). Dulbecco's modified eagle medium (DMEM) and fetal bovine serum (FBS) were obtained from Invitrogen (Invitrogen, Paisley, UK). DMSO (Dimethyl sulfoxide) and Tris-EDTA (Tris-Ethylene Diamine Tetraacetic Acid) were purchased from Applichem (Applichem, Darmstadt, Germany). DMEM-Hepes, PBS (Phosphate Buffer Saline), FBS (Fetal Bovine Serum), Trypsin–EDTA, and DMSO reagents were purchased from Invitrogen (Invitrogen, Darmstadt, Germany). The stock solution was filtered through a 0.22 μM syringe filter, then aliquoted and stored in the dark at room temperature.

Kras mutation detection

All patient tumor tissue specimens were snap-frozen and preserved in liquid nitrogen before use. Paraffin sections were then prepared and one section from each case was chosen for Hematoxylin-Eosin staining. An experienced pathologist analyzed the Hematoxylin-Eosin section to confirm the presence of tumor tissue. Subsequently, tissue samples from at least five serial sections were macrodissected to ensure that the specimens contained at least 80% tumor cells. DNA was isolated from these tissue samples using the Qiagen DNA extraction Kit (Qiagen, Berlin, Germany) according to manufacturer instructions. Subsequently, mutations in codon 12 of K-ras exon 1 were detected in these genomic DNA samples by PCR-based direct sequencing (Supplementary Table S2). The PCR primers for K-ras used were K-ras-Forward: 5′-AAGGCCTGCTGAAAGTGACTG-3′ and K-ras-Reverse: 5′-CTGGTGCAGGACCATTCTTCAG-3′. The PCR reaction was carried out in a total volume of 50 μl containing 150 ng of the extracted genomic DNA. PCR amplification conditions were as follows: initial denaturation at 94°C for 1 min, followed by 40 cycles of denaturation at 94°C for 30 s, annealing at 55°C for 30 s, and extension at

72°C for 30 s, with a final extension step at 72°C for 5 min in a TP-600 thermal cycler (TaKaRa). PCR products were then analyzed using 2.5% agarose gel electrophoresis and purified for further direct DNA sequencing through an ABI-3730XL DNA Analyzer (Applied Biosystems, Europe).

MDSC experiments

For MDSCs analysis, single-cell suspensions were prepared from lymph nodes, lungs and tumour tissues. Bone marrow cells were obtained by flushing bones with PBS using a 28G 1/2 syringe. Tumour and lung tissues were dissociated and digested with collagenase for 50min at 37C. Erythrocytes were lysed with ACK lysis buffer (Gibco). These cells were labelled with fluorescence-conjugated Ly6C (dilution 1/300), Ly6G (dilution 1/150) and CD11b (dilution 1/250) antibodies from Biolegend and analysed on a FACS flow cytometer (BD Biosciences). Different subsets of MDSCs were sorted with a FACS Aria cell sorter (BD Biosciences). For co-culture experiments, tumour cells were cultured either alone (control) or with 0.5 mM CFSE labelled MDSCs in 10% FBS RPMI media for 24–48 h at the ratio of 1:1. After incubation, FITC negative tumour cells were isolated with a FACS Aria cell sorter for following experiments. Culture supernatant were collected for cytokine analysis.

PBMC cell isolation

Blood samples were collected from lung cancer patients and healthy controls (HC). Peripheral blood mononuclear cells (PBMC) were isolated with Ficoll–Hypaque by density gradient centrifugation within 1 hour of sample collection. Fresh tumor tissues were obtained from lung cancer patients during surgical resection. Next, samples were minced and digested with type I collagenase in RPMI 1640. Digested cells were filtered through a nylon mesh (60 mm) and washed with PBS. $CD4^{+}TIGIT^{+}$/$CD4^{+}TIGIT^{-}$ and $CD8^{+}TIGIT^{+}$/ $CD8^{+}TIGIT^{-}$ cells were sorted and analyzed using a BD FACS Aria (BD Biosciences, Germany).

Immunostaining for Flow Cytometry

For surface immunostaining, cells were stained with the following antibody to markers: CD25, CD45.2, CD4, CD8, CD11b, F4/80, CD11c, Ly6C, and Ly6G. For intracellular STING staining, after surface staining, cells were fixed and permeabilized. Rabbit unconjugated STING antibody and Rabbit immunoglobulin G isotype control (Invitrogen, Carlsbad, CA) were used as primary antibody, and AF488 conjugated goat anti-rabbit used as secondary antibody (Life Technologies, Carlsbad,

CA). Cells were run on LSRII (BD Biosciences, San Jose, CA) and analyzed with FlowJo software (Treestar Inc, Ashland, OR).

Immunofluorescence analysis

Cells were fixed in 4% formaldehyde for 15 min at room temperature prior to cell permeabilization with 0.1% Triton X-100 (4°C, 10 min). Cells were saturated with PBS containing 2% BSA for 1 h at room temperature and processed for immunofluorescence with Ly6C, Ly6G, Ki67 and TNF-a antibodies, respectively, at 4ºC overnight. Then, they were incubated with Alexa Fluor 488-conjugated anti-rabbit IgG antibody (1:100). Between all incubation steps, cells were washed three times for three minutes with PBS containing 0.2% BSA. Fluorescence signals were analyzed using a Carl Zeiss fluorescent microscope at a 100× magnification, with excitation and emission wavelengths of 488 nm and 520 nm, respectively, using image analysis software.

Transwell migration assay

Cell invasion was evaluated using a matrigel invasion chamber. The migration assay was conducted in a 24-well transwell cell culture apparatus fitted with multiporous polycarbonate membrane insert (8-μm pore size) (Corning). In brief, H1299/H1975 cells were collected and resuspended in serum free media at a density of 1×10^5 cells/ml. The top chamber of transwell was loaded with 100 μl of cell suspension and the lower chamber was filled with 0.5 ml of media supplemented with 10% FBS as a chemo attractant. After incubation at 37 °C in 5% CO_2 for 24 h, the filters were removed, rinsed two times with PBS, fixed with methanol and stained with 0.5% crystal violet for 20 min. Cells on the upper side of the filter were wiped off with cotton swabs. The cells that migrated on the lower side of the filter were determined by counting specified cross-sectional fields on the filter with a phase-contrast microscope. The experiments were performed in triplicates.

T-cell function assays

For T-cell activation assays, cells were seeded in 96-well plates and stimulated with anti-CD3/CD28 Dynabeads (aCD3/CD28) for 12 hours to measure CD69 expression by flow cytometry. For cell proliferation assay, cells were labeled with carboxyfluorescein. diacetate succinimidyl ester (CFSE) and stimulated with aCD3/CD28 at 37C with 5% CO2 for 4 days. Cells were collected, and the dilution of intracellular CFSE caused by proliferation was calculated using a flow cytometer (30).

For intracellular cytokine stimulation assays, cells were stimulated with 500 ng/mL PMA and 1 mg/mL ionomycin (Sigma-Aldrich) for 5 hours at 37C with 5% CO2. Cells were collected and stained with V450-conjugated anti-IFNg and PE-Cy7–conjugated anti-TNFa antibodies.

Isolation of IL-10-producing cells

$CD4^{+}CD25$- T cells were isolated from TILs of patients with lung cancer by magnetic sorting as previously reported. Briefly the non-$CD4^{+}$ cells were removed by a magnetically labeled cocktail of antibodies followed by depletion of $CD25^{+}$ cells (Invitrogen). CD4+$CD25^{-}$T cells were stimulated with Dynabeads that were coupled to anti-CD3(OKT-3, Biolegend) alone or in combination with anti-CD46 (Biolegend) or anti-ICOS(ISA-3; Invitrogen) antibodies using the Dynabeads antibody coupling kit (Invitrogen). The bead to cell ratio was 0.4:1. Cells were cultured in the presence of 250 U/mL IL-2 (Abcam) for 24–48 h. Then, cells were magnetically sorted into IL-10^{low} and IL- 10^{high} fractions using the secretion assay-cell enrichment kit from Invitrogen.

TUNEL assay

TUNEL staining was performed to detect in situ DNA fragmentation as a marker of apoptosis using an In Situ Apoptosis Detection Kit (Takara Bio, Otsu, Japan). Following treatment, LC-DR cells were fixed and permeabilized and cells were reacted with terminaldeoxynucleotidyl transferase and fluorescein isothiocyanate (FITC)-deoxyuridine triphosphate for 90 min at 37 °C. FITC signals were visualized using a Carl Zeiss confocal laser microscope.

Annexin V apoptosis assay

Cellular apoptosis was analyzed using the FITC Annexin V/PI Apoptosis assay (BD Biosciences Germany) according to manufacturer instructions. Briefly, after undergoing serum deprivation, cells were harvested, washed with PBS, and resuspended in 1 × binding buffer at a concentration of 1×10^6 cells/mL. To 100 μL of the cell suspension, 5 μL of Annexin V-FITC and 2.5 μL of PI (250 μg/mL) were added. After incubation for 15 min, the cells were analyzed by fluorescence microscopy and flow cytometry using a FACSCalibur flow cytometer and data were analyzed using CellQuest software (BD Biosciences, San Jose, CA). Living cells (Annexin V −/PI −), early apoptotic cells (Annexin V +/PI −), late apoptotic cells (Annexin V +/PI +), and necrotic cells (Annexin V −/PI +) were distinguished. A minimum of 5000 events were collected on each sample by duplicate.
