# Supplementary material for: Kras-driven intratumoral heterogeneity triggers infiltration of M2 polarized macrophages via the circHIPK3/PTK2 immunosuppressive circuit
Source: Sci Rep. 2021 Jul 29;11:15455. doi: 10.1038/s41598-021-94671-x (PMC8322174; doi:10.1038/s41598-021-94671-x)
Supplement: Supplementary file 2 — Supplementary Table S1. [file 41598_2021_94671_MOESM2_ESM.pdf]

Clinical Characteristics	Mean Age	Sex		Histological Type		
		Male	Female	Squamous cell carcinoma	Adeno-carcinoma	Large cell carcinoma
Number of patients	67	56 (59)*	40 (41)	37 (39)	29 (30)	30 (31)

Table S1. Clinical characteristics of lung cancer patients. (*) Numbers in parenthesis indicate percentiles (%) in the total population of patients.
