# Supplementary material for: Kras-driven intratumoral heterogeneity triggers infiltration of M2 polarized macrophages via the circHIPK3/PTK2 immunosuppressive circuit
Source: Sci Rep. 2021 Jul 29;11:15455. doi: 10.1038/s41598-021-94671-x (PMC8322174; doi:10.1038/s41598-021-94671-x)
Supplement: Supplementary file 3 — Supplementary Table S2. [file 41598_2021_94671_MOESM3_ESM.pdf]

KRAS codon 12 Mutation Status							
**Clinical Features**	**n**	**Gly12Cys (G12C)**	**Gly12Val (G12V)**	**Gly12Asp (G12D)**	**Gly12Arg (G12R)**	**Gly12Ala (G12A)**	**Gly12Ser (G12S)**
**Stage I**	24	10(42)	4(17)	4(17)	3(12)	1(4)	2(8)
**Stage II**	26	12(46)	5(19)	3(12)	3(12)	2(8)	1(4)
**Stage III**	42	17(40)	9(21)	5(12)	5(12)	3(7)	3(7)
**Stage IV**	32	19(60)	5(16)	3(9)	2(6)	2(6)	1(3)
**Chemoresistant**	51	28(55)	12(23)	4(8)	3(6)	1(2)	3(6)
**Chemosensitive**	45	11(24)	9(20)	8(18)	7(16)	6(13)	4(9)
**Metastatic**	47	32(68)	8(17)	3(6)	2(4)	1(2)	1(2)
**Non-metastatic**	49	12(24)	11(22)	8(16)	9(18)	5(10)	4(8)

Table S2. Correlation between KRAS mutation status in lung cancer patients. Numbers in parenthesis indicate percentiles (%) in the total population of patients.
