# Supplementary material for: Kras-driven intratumoral heterogeneity triggers infiltration of M2 polarized macrophages via the circHIPK3/PTK2 immunosuppressive circuit
Source: Sci Rep. 2021 Jul 29;11:15455. doi: 10.1038/s41598-021-94671-x (PMC8322174; doi:10.1038/s41598-021-94671-x)
Supplement: Supplementary file 4 — Supplementary Table S3. [file 41598_2021_94671_MOESM4_ESM.pdf]

Gene	Primer
circPTK2	Forward: 5′- AATCGTGGTGAACCCATAGTG -3′<br>Reverse: 5′- CTGCAAGCATTAGCATCCCT -3′
circHIPK3	Forward: 5′- TATGTTGGTGGATCCTGTTCGGCA -3′<br>Reverse: 5′- TGGTGGGTAGACCAAGACTTGTGA -3′
IFN-γ	Forward: 5′- GGCCATCAGCAACGGCGT-3′<br>Reverse: 5′- TGGGTTGTTGACTTGGC-3′
GATA-3	Forward: 5′- GTCTGCAGCCAGGAGAGC -3′<br>Reverse: 5′- ATGCATCAAACAACTGTCA -3′
GAPDH	Forward: 5'-ACCACAGTCCATGCCATCAC-3'<br>Reverse: 5'-TCCACCACCCTGTTGCTGT-3'

Table S3. List of the gene primers used to perform mRNA analysis for the quantitative polymerase chain reaction (qRT-PCR).
