# Supplementary material for: Kras-driven intratumoral heterogeneity triggers infiltration of M2 polarized macrophages via the circHIPK3/PTK2 immunosuppressive circuit
Source: Sci Rep. 2021 Jul 29;11:15455. doi: 10.1038/s41598-021-94671-x (PMC8322174; doi:10.1038/s41598-021-94671-x)
Supplement: Supplementary file 5 — Supplementary Table S4. [file 41598_2021_94671_MOESM5_ESM.pdf]

$CD4^+/CD8^+/Foxp3^+$ Lymphocytic Infiltration Status							
**Clinical Features**	**n**	**$CD4^-$ / $CD8^-$**	**$CD4^+$ / $CD8^-$**	**$CD4^-$ / $CD8^+$**	**$CD4^+$ / $CD8^+$**	**$CD4^+$/$Foxp3^+$**	**$CD8^+$ / $Foxp3^+$**
**Stage I**	24	4 (17)	18 (75)	23 (96)	22 (92)	19 (79)	17 (71)
**Stage II**	19	2 (11)	15 (79)	17 (88)	15 (79)	18 (94)	14 (74)
**Stage III**	21	4 (19)	11 (52)	13 (62)	12 (57)	10 (48)	11 (52)
**Stage IV**	32	3 (9)	7 (22)	5(16)	4 (12)	6 (19)	2 (12)
**Chemoresistant**	51	14 (27)	5 (10)	7 (14)	5 (8)	9 (18)	7 (14)
**Chemosensitive**	45	22 (49)	25 (56)	31 (69)	36 (80)	41 (91)	39 (87)
**Metastatic**	47	7 (15)	11(23)	14 (30)	12 (26)	11 (23)	13 (28)
**Non-metastatic**	49	27 (55)	32 (65)	28 (62)	37 (57)	41 (84)	35 (71)

Table S4. Correlation between combined $CD4^+/CD8^+/Foxp3^+$ lymphocytic infiltration status in lung cancer patients. Numbers in parenthesis indicate percentiles (%) in the total population of patients.
